# Hepatitis B virus infection among pregnant women in Taiwan: Comparison between women born in Taiwan and other southeast countries

**DOI:** 10.1186/1471-2458-8-49

**Published:** 2008-02-07

**Authors:** Ching-Chiang Lin, Hsiu-Shu Hsieh, Yu-Jie Huang, Yeou-Lih Huang, Ming-Kun Ku, Hsin-Chia Hung

**Affiliations:** 1 Department of Laboratory Medicine, Fooyin University Hospital, Pingtong, Taiwan; 2 Basic Medical Science Education Center, Fooyin University, Kaohsiung, Taiwan; 3 Graduate Institute of medicine, College of Medicine, Kaohsiung Medical University, Kaohsiung, Taiwan; 4 Department of Nursing, Tri-Service General Hospital, Taipei, Taiwan; 5 Department of Radiation Oncology, Chang Gung Memorial Hospital – Kaohsiung Medical Center, Chang Gung University College of Medicine; Kaohsiung, Taiwan; 6 Faculty of Biomedical Laboratory Science, Kaohsiung Medical University, Kaohsiung, Taiwan; 7 Department of Internal Medicine Fooyin University Hospital, Pingtong, Taiwan; 8 Graduate Institute of Health Care, MeiHo Institute of Technology, Pingtong, Taiwan; 9 Global Center of Excellence for Oral Health Research and Development, Kaohsiung Medical University, Kaohsiung, Taiwan

## Abstract

**Background:**

Taiwan's national vaccination program has successfully decreased the prevalence of hepatitis B infection after twenty years of implementation and might be indirectly beneficial to the second generation. In this study, we compared the hepatitis B infection status of two groups: pregnant Taiwanese women and other Southeast Asian women, who because they had immigrated later in life to Taiwan by marriage to a Taiwanese man, had not been exposed to that vaccination program to evaluate the effect of hepatitis vaccination program on women of child-bearing age and further explored the potential impact of immigration on the hepatitis B public health policy in Taiwan.

**Methods:**

Data was collected from 10,327 women born in Taiwan and 1,418 women born in other Southeast Asian countries, both groups receiving prenatal examinations at Fooyin University Hospital between 1996 and 2005. The results of serum hepatitis B s-Antigen (HBsAg) and hepatitis B e-Antigen (HBeAg) tests and other demographic data were obtained by medical chart review.

**Results:**

The pregnant women from Taiwan had a higher HBsAg positive rate (15.5%) but lower HBeAg(+)/HBsAg(+) ratio (32.1%) than the women from other countries (8.9% and 52.4%). For those born before July, 1984, the period of no national vaccination program, Taiwanese women had a higher HBsAg positive rate than other Southeast Asian women (15.7% vs. 8.4%), but for women born after that day and before June 1986, the period of vaccination for high risk newborns, the HBsAg positive rates found to be slightly lower for Taiwanese women than for other Southeast Asian women (11.4% vs. 12.3%) and the difference was more significant (3.1% vs. 28.6%) after June 1986, the period of vaccination for all newborns. While the HBeAg(+)/HBsAg(+) ratios decreased with age in both groups, they were consistently higher in women from other Southeast Asian countries than in women born in Taiwan after age 20.

**Conclusion:**

In Taiwan, the neonatal vaccination program that was implemented in 1984 has successfully reduced hepatitis B infection among pregnant women in present day, and is likely to indirectly prevent hepatitis B infection in the next generation. However, the increasing number of pregnant women from other Southeast Asian countries without a national neonatal vaccination program or with a program that was introduced later than the one in Taiwan will likely lessen the positive impact of this program and should be further assessed.

## Background

There are estimated 350 million people infected with Hepatitis B Virus (HBV) worldwide. While prevalence varies widely, it is especially high in Taiwan and other Asian countries [1]. HBV has been associated with hepatocellular carcinoma, the second most common cancer, and cirrhosis and chronic hepatitis, the seventh leading cause of death in Taiwan in 2004 [2-4].

Based on the results of Beasley et al's randomized blind controlled trial demonstrating the effectiveness of hepatitis B vaccine in preventing perinatal HBV infection, Taiwan became one of the first countries to introduce a national HBV vaccination program in July 1984 [4,5]. During the first two years of the program, only newborns born to mothers found positive for HBsAg received vaccinations; after June, 1986, coverage was further extended to all newborns [4]. The coverage rates of HBV vaccine for the target population were all more than 90% since the initiation of the program in 1984 [4]. This program has successfully reduced the prevalence of hepatitis B in children and the incidence of hepatocellular carcinoma in those exposed to the vaccine [1,6,7]. After two decades, with these vaccinated women now at child-bearing age, the benefits of this vaccination program may soon be seen in the second generation.

One possible threat to the completeness of is program is recent interest of Taiwanese men in marrying women from Southeast Asian countries, where prevalence of hepatitis B is also high but there has been no similar hepatitis vaccination program in place [8]. This study investigated hepatitis B infection status among pregnant Taiwanese women and pregnant women from five Southeast Asian countries across three periods of different hepatitis B vaccine programs in Taiwan (no vaccination, vaccination for high risk newborns and vaccination for all newborns) to evaluate the effect of hepatitis B vaccination program on women of child-bearing age in Taiwan and further explore the potential impact of immigration on the Hepatitis B public health policy in Taiwan.

## Methods

We recruited 11,745 pregnant women, 10,327 women from Taiwan and 1,418 women from five Southeast Asian countries (Philippines, Cambodia, Indonesia, Thailand, and Vietnam), all receiving routine prenatal examinations at Fooyin University Hospital between 1996 and 2005. This regional hospital provides obstetric health care to several townships in Pingtong, Taiwan.

Blood samples were obtained from all participants during the early second trimester and tested for the hepatitis B s-Antigen (HBsAg) and hepatitis B e-Antigen (HBeAg). The serologic antigens were analyzed by Microparticle Enzyme Immunoassay (MEIA) method using AxSYM (Abbot Laboratories, Chicago, Illinois, USA) HBsAg (V2) and HBeAg 2.0 kits. Data on age, nationality, and HBsAg and HBeAg tests were collected retrospectively by reviewing the medical records of these women. This protocol for study was approved by Fooyin University Hospital's IRB. All personal information was removed, making it impossible to link a participant's individual information without further permission from the hospital.

The participants were first categorized by nationality groups and sub-categorized by specific time periods. Age was expressed as mean ± 1SD for women from Taiwan and the five other countries. We compared the differences between women from Taiwan and other five countries using chi-square test or fisher's exact test for those with small sample size, and calculated the odds ratio (OR) for a positive HBsAg rate for these women and the OR for a positive HBeAg rate among HBsAg(+) women. We further conducted the analyses stratified by age and date of birth. In order to compare the HBsAg positive rates over time and under different coverage strategies (no vaccination, vaccination for newborns of high risk and vaccination for all newborns), we stratified our data by relevant time periods. The p-value < 0.05 was considered as significant. All statistical operations were performed using SAS software (version 9.12 ; SAS Institute, Cary, NC).

## Results

From 1996 to 2005, 11,745 pregnant women, including 10,327 Taiwanese women, 1,114 from Vietnam, 171 from Indonesia, 71 women from the Philippines, 42 from Cambodia, and 20 from Thailand received routine prenatal examinations at Fooyin University Hospital. The number and percentages of pregnant Southeast Asian women seeking prenatal examinations in this hospital increased from 50 women (1.2% of the total) between 1996 and 1999 to 919 (24.6% of the total) between 2003 and 2005 (Table 1). The average prevalence rate of hepatitis B among Taiwanese women was 15.5% and among Southeast Asian women 8.9% (9.5% for Cambodia, 9.0% for Philippines, 8.9% for Vietnam, 8.8% for Indonesia, and 5.5% for Thailand).

**Table 1:** Numbers and HBsAg positive rates of pregnant women born in Taiwan and other six Southeast Asian countries from 1996 to 2005

	Taiwan	Other South East Asia countries					
	
		All countries	Philippines	Cambodia	Indonesia	Thailand	Vietnam
		
Number of women							
1996–1999	4206	50	24	3	12	5	6
2000–2002	3305	449	33	23	87	6	300
2003–2005	2816	919	14	16	72	9	808
Total	10327	1418	71	42	171	20	1114
Number of HBsAg(+) women							
	1597	126	7	4	15	1	99
Percentage of women with HBsAg(+)							
	15.5%	8.9%	9.9%	9.5%	8.8%	5.0%	8.9%

Women born in Taiwan appeared to be older than the women from the five other countries (26.0 years vs. 23.4 years). Taiwanese women also had significantly higher HBsAg positive rates (15.5% vs. 8.9%) but lower HBeAg(+)/HBsAg(+) ratios (32.1% vs. 52.4%). Table 2 presents the differences in HBsAg positive rates between Taiwanese women and other Asian women stratified by age and date of birth. Taiwanese women seemed to have higher HBsAg positive rates (15.5% vs. 8.9%). However, the declining trend in seropositive rates over age and time period was only observed in Taiwanese women, for whom, the rates decreased from 16.6 % for the women aged >30 years to 12.8% for those aged ≦20 years, and decreased from 15.7% for women born before July 1984, 11.4% for those born between July1984 and June 1986, to 3.1% for those born after June 1986. Taiwanese women born after implementation of the vaccine program had lower HBsAg positive rate than women born outside of Taiwan (8.8% vs. 13.1%). For those born after June 1986, the date of initiation of coverage for all newborns, the seropositive rate among Taiwanese women even decreased into 3.1% while the rate in women from the five other countries was 28.6% and the odds ratio was 12.4 (95% CI: 1.67–91.9) with p-value of 0.04.

**Table 2:** Age-specific prevalence of HBsAg (+) among pregnant women born in Taiwan and other Southeast Asia countries

	Taiwan	Southeast Asia	Southeast Asia vs. Taiwan	
	
	HBsAg(+)/Total (%)	HBsAg(+)/Total (%)	OR (95% CI)	P-value
Total	1597/10327 (15.5 %)	126/1418 (8.9 %)	0.53 (0.44–0.65)	<0.001

Age (year)				
≦20	173/1354 (12.8 %)	37/332 (11.1 %)	0.86 (0.59–1.25)	0.420
>20–≦25	579/3705 (15.6 %)	60/760 (7.9 %)	0.46 (0.35–0.61)	<0.001
>25–≦30	530/3372 (15.7 %)	22/245 (9.0 %)	0.53 (0.34–0.83)	0.005
>30	315/1896 (16.6 %)	7/81 (8.6 %)	0.48 (0.22–1.04)	0.063
Date of birth				
Before July 1984	1570/10021 (15.7 %)	107/1273 (8.4 %)	0.49 (0.40–0.61)	<0.001
before 1970	285/1909 (14.9 %)	3/48 (6.3 %)	0.38 (0.12–1.23)	0.11
1970- before 1978	900/5335 (16.9 %)	34/310 (11.0 %)	0.61 (0.42–0.87)	0.007
1978- before July 1984	385/2777 (13.9 %)	70/915 (7.7 %)	0.52 (0.39–0.67)	<0.001
July, 1984 and after	27/306 (8.8 %)	19/145 (13.1 %)	1.56 (0.84–2.91)	0.163
July, 1984 – June, 1986	24/210 (11.4 %)	17/138 (12.3 %)	1.09 (0.56–2.11)	0.80
July, 1986 and after	3/96 (3.1 %)	2/7 (28.6 %)	12.4 (1.67–91.87)	0.04^a^

^a ^: P-value of Fisher's exact test

Table 3 presents the differences in HBeAg(+)/HBsAg(+) ratios between Taiwanese women and other Asian women stratified by age and date of birth. Taiwanese women had significantly lower HBeAg(+)/HBsAg(+) ratios (32.1% vs. 52.4%) than other Asian women. The HBeAg(+)/HBsAg(+) ratios declined over age, from 54.3% for Taiwanese women aged ≦20 years to 19.5% for those aged >30 years and 56.8% for Southeast Asian women aged ≦20 years to 28.6% for those age>30 years. Except for women 20 years old or below, women with positive HBsAg results from the five other countries seemed to have a higher HBeAg positive rate than Taiwanese women, though the statistical significance was only reached for the women aged >25–≦30 years or the women born before July, 1984.

**Table 3:** Age-specific prevalence of HBeAg (+) among HBsAg(+) women born in Taiwan and other Southeast Asia countries

	Taiwan	Southeast Asia	Southeast Asia vs. Taiwan	
	
	HBeAg(+)/HBsAg(+) (%)	HBeAg(+)/HBsAg(+) (%)	OR (95% CI)	P-Value
Total	513/1597 (32.1 %)	66/126 (52.4 %)	2.32 (1.61–3.35)	<0.001

Age (year)				
≦20	94/173 (54.3 %)	21/37 (56.8 %)	1.10 (0.54–2.26)	0.789
>20–≦25	219/579 (37.8 %)	29/60 (48.3 %)	1.54 (0.90–2.62)	0.114
>25–≦30	139/530 (26.2 %)	14/22 (63.6 %)	4.92 (2.02–11.99)	<0.001
>30	61/315 (19.4 %)	2/7 (28.6 %)	1.67 (0.32–8.79)	0.548
Date of birth				
Before July, 1984	496/1570 (31.6 %)	54/107 (50.5 %)	2.21 (1.49–3.27)	<0.001
before 1970	56/285 (19.6 %)	0/3 (0 %)	--	--
1970- before 1978	274/900 (30.4 %)	22/34 (64.7 %)	4.19 (2.04–8.58)	<0.001
1978- before July 1984	166/385 (43.1 %)	32/70 (45.7 %)	1.11 (0.67–1.85)	0.687
July, 1984 and after	17/27 (63.0 %)	12/19 (63.2 %)	1.01 (0.30–3.40)	0.989
July, 1984 – June, 1986	14/24 (58.3 %)	10/17 (58.8 %)	1.02 (0.29–3.60)	0.975
July, 1986 and after	3/3 (100 %)	2/2 (100 %)	-	-

## Discussion

In this study, we found that the positive rate of HBsAg among pregnant Taiwanese women to be significantly higher than that of the pregnant women from the five other Southeast Asian countries, but the difference disappeared in those born in and after July, 1984. The HBeAg positive rates in women with HBsAg seropositivity appeared to be higher in women from other countries than in Taiwanese women, especially for those aged of >25 to ≦30 years.

The national vaccination program has successfully prevented the vertical transmission from mother to child and reduced the incidence and mortality of hepatocellar carcinoma in Taiwan [1,6,7,9-11]. In that program, infants were vaccinated with four doses of plasma-derived vaccine at 0, 1, 2, and 12 months. In Nov. 1992 the plasma-derived vaccine was replaced by three-dose recombinant yeast vaccine at ages 0, 1 and 6 months. At the same time, all pregnant women were screened for HBsAg, and women with HBsAg positive serum were further tested for HBeAg. Newborns of highly infectious carriers mothers, who were found positive for both HBsAg and HBeAg, further received hepatitis B immune globulin at birth [1]. After 15 years, the prevalence of HBsAg among persons younger than fifteen years old dropped from 9.8% in 1984 to 0.7% in 1999[9]. Studies have also reported the incidence of hepatocellular carcinoma among the children born after the initiation of vaccination to be significantly lower than those born before 1984 and have suggested that the program has further prevented liver cancer induced by HBV [6].

Our findings further suggest that the nationwide vaccination program is not only just successful at preventing mother-to-child infection but might also have started to benefit the second generation indirectly through by reducing the rates of maternal carriers. The pregnant women born in July, 1984 or after were at lower risk of HBV infection and, therefore, less likely to continue the vertical transmission than those born before July, 1984. This decreasing trend was found in Taiwan women only in this study and, hence, women born in other Southeast Asian countries could serve as a natural comparison group representing those who had not received vaccinations at birth in this study. While the Southeast Asian sample had increase in HBsAg positive rates (8.4% to 13.1%), the Taiwanese women had drop (15.7% to 8.8%). We further stratified our data by the time periods representing different coverage strategies (July, 1984 to June 1986, covering newborns of HBV carriers and July, 1986 and after, covering all newborns) and found an 11.4% HBV infection rate in women born during the earlier period, and found a 3.1% HBV infection rate in women born during that later period. Although this finding might be confounded by age, it would support the idea that coverage of all newborns is more effective than coverage for newborns of HBV carrier mothers only.

In several low endemic regions, the immigrants from other highly endemic regions have been reported to have high prevalence of HBsAg, suggesting that the immigration patterns might introduce the challenges from hepatitis B infection in those countries. In the United Kingdom, for example, a survey of 117 Chinese residents and 234 non-Chinese controls reported the 12.8% of Chinese and 0.4% non-Chinese residents were HBsAg positive persons [12]. In France, an investigation of pregnant women screened for HBsAg found that positive results in 0.29% of the women of French origin, 7.15% of Southeast Asian origin, and 6.52% for Sub Saharan African origin [13]. In the USA, that HBsAg positivity was found in 5.8% of the Asians (11.1% for Cambodian and 10.4% for Chinese), 1.0% of black non-Hispanics, 0.6% of white non-Hispanics and 0.1% of Hispanics, suggesting that immigration and migration patterns might have a detrimental impact on public health with regard to HBV infection [14].

The difference in HBV infection rates in Taiwan and other Southeast Asian countries are not as significant as those from low endemic regions like in USA and France, and their immigrants. In fact, these pregnant Southeast Asian women even had slightly lower HBsAg positive rate than the Taiwanese women. However, the Southeast Asian women in this study might not have received vaccinations in the same period in their countries of origin, as reflected in the higher prevalence of HBsAg among these Southeast Asian women than that of among Taiwanese women born in 1984 or after. Hence, the increasing number of pregnant women from other Southeast Asian countries, making up1.2% of our total in 1996–1999 and 24.6% in 2003–2005, might attenuate the indirect beneficial effects of vaccination program on the second generation.

Moreover, we found the HBeAg positive rates seemed to be significantly higher in the Southeast Asian women (52.4%) who tested positive for HBsAg than in Taiwanese women (32.1%). HBeAg is a seromarker related to infective HBV particles and its seropositivity might represent a high level of viral replication in hepatocytes. Those, who are positive for HBsAg and seropositive for HBeAg have been reported to be more likely to develop hepatocellar carcinoma and chronic liver disease [15-17]. Therefore, the presence of the less spontaneous HBeAg seroconversion among these Southeast Asian women might suggest that they are more likely to develop severe liver disease in the future. Furthermore, it would suggest that an increasing number of newborns are being born to highly infectious maternal carriers, who have been tested positive for both HBeAg and HBsAg. The newborn children of these women would not only need to be administered expensive hepatitis B immune globulin at birth but they are also less protected by the vaccination program than the children born to low infectious maternal carriers [1,7]. Recently, several antiviral therapies, including interferon and Lamivudine, have been proved to effectively reduce the serum HBV DNA levels and increase the HBeAg seroconversion rates [18,19]. Hence, in addition to using the HBV vaccine to reduce the hepatitis B infection to prevent the severe liver diseases and vertical transmission at primary level, early screening of these migrant women and other high risk populations can help identify high-risk patients who might benefit from antiviral treatment before pregnancy.

The potential modifiable factors associated with the different rates in HBeAg seroconversion is also worthy of further exploration. One previous study in Taiwan reported that the mean ratios of HBeAg/HBsAg declined steadily from 1993 to 2000, with the mean average annual declines in HBeAg rate being 1.2%, 0.6%, 0.4% and 0.1% in those <26 years, 26–30 years, 31–35 years and >35 years, respectively [20]. The reason for this secular trend was still unclear, since there were no related health programs being performed during this period.

Age and treatment of HBV might be associated with HBeAg seroconversion but neither of them seemed to be able to explain the differences in seroconversion rates in this study [15,17,20]. Although the HBeAg positive rates decreased with age, the age-specific HBeAg positive rates for the Southeast Asian women were consistently higher than those of the Taiwanese women above the age of 20 years. During prenatal examination, none of these pregnant women reported have received any previous treatment for hepatitis B infection.

The genotypes of hepatitis B virus might be related to HBeAg positive rates. HBV can be classified into several genotype (A, B, C, D, E, F and G), which often vary in different geographical areas [21-23]. The dominant genotypes in Taiwan and the five Southeast Asian countries in this study are B and C [21,24]. It has been suggested that genotype C patients have a have a longer delay in HBeAg seroconversion in the immune clearance phase of chronic HBV infection than genotype B patients [25,26]. Further analysis is needed to explore the possible association between genotype and different HBeAg seroconversion rates in these populations.

One limitation of this study is that we did not collect individual histories of vaccination and blood samples for testing antibodies to HBsAg and HBcAg. Hence, we could not directly analyze the influence of vaccination or efficacy of vaccination on HBsAg and HBeAg status at individual level. Instead, we considered Taiwanese women born during the period vaccination program was being vaccinated, since the coverage rates at that time were more 90% [4]. The countries from which other groups of pregnant women arrived waited until a much later to implement vaccination programs. For example, Vietnam introduced the vaccine in pilot areas in 1997 and nationally in 2003 and Cambodia implemented the program in four health centers only in 2001 but not yet nationally until 2005 [8]. Furthermore, the misclassification of vaccine experience is more likely to attenuate the findings of this study.

That the tests for HBsAg and HBeAg were performed in the prenatal examination is another limitation. There is possibility that these women from other countries might have been infected after moving into Taiwan since they have lived in Taiwan for a certain period. However, the prevalences of HBsAg among women in this study were similar to other reports conducted in these countries, suggesting that this limitation should not introduce appreciable bias to our results. The findings of this study and other studies were 9.9% vs. 2.0–16.5% for Philippines, 9.5% vs. 9.1% for Cambodia, 8.8% vs. 6.2 – 8.8% for Indonesia, 5.0% vs. 8.2%–10.3% for Thailand, and 8.9% vs. 9–16% for Vietnam [27-35].

## Conclusion

The lower HBsAg positive rate among Taiwanese women born after July, 1984 suggests that the vaccination program has effectively reduced the hepatitis B infection among Taiwan women of child-bearing age and further started to indirectly prevent hepatitis B infection in the second generation, compared with women from other Southeast Asian countries. These immigrants also had higher HBeAg(+)/HBsAg(+) ratios, which might result in more severe health outcomes and more expensive but less efficient vaccination program. Hence, the increasing number of foreign brides from Southeast Asian countries will likely lessen the positive impact of this vaccination program and should be further assessed.

## List of abbreviations

HBV: hepatitis B virus; HBsAg: hepatitis B s-Antigen; HBeAg: hepatitis B e-Antigen

## Competing interests

The author(s) declare that they have no competing interests.

## Authors' contributions

MKK and HSH were in charge of data collection. YJH and YLH helped with study design and data interpretation. CCL and HCH analyzed the data and completed the fist draft. All authors provided comments and assisted in completing the final version of this manuscript.

## Pre-publication history

The pre-publication history for this paper can be accessed here:


